# Oxidative Stress and Treg and Th17 Dysfunction in Systemic Lupus Erythematosus

**DOI:** 10.1155/2016/2526174

**Published:** 2016-08-11

**Authors:** Ji Yang, Xue Yang, Hejian Zou, Ming Li

**Affiliations:** ^1^Department of Dermatology, Zhongshan Hospital, Fudan University, Shanghai 200032, China; ^2^Division of Rheumatology, Huashan Hospital, Fudan University, Shanghai 200040, China; ^3^Institute of Rheumatology, Immunology and Allergy, Fudan University, Shanghai 200040, China

## Abstract

Systemic lupus erythematosus (SLE) is an autoimmune disease that involves multiple organ systems. The pathogenic mechanisms that cause SLE remain unclear; however, it is well recognized that the immune balance is disturbed and that this imbalance contributes to the autoimmune symptoms of SLE. Oxidative stress represents an imbalance between the production and manifestation of reactive oxygen species and the ability of the biological system to readily detoxify the reactive intermediates or to repair the resulting damage. In humans, oxidative stress is involved in many diseases, including atherosclerosis, myocardial infarction, and autoimmune diseases. Numerous studies have confirmed that oxidative stress plays an important role in the pathogenesis of SLE. This review mainly focuses on the recent research advances with respect to oxidative stress and regulatory T (Treg)/helper T 17 (Th17) cell dysfunction in the pathogenesis of SLE.

## 1. Introduction

Systemic lupus erythematosus (SLE) develops following an encounter by genetically predisposed people to environmental agents, such as ultraviolet light (UV), infections, and cigarette smoke, that cause oxidative stress, but how this oxidative damage modifies the immune system to induce lupus flares remains unknown [1–3]. Activation, proliferation, or death of cells of the immune system is dependent on the production of reactive oxygen intermediates (ROI) and ATP synthesis in the mitochondria [4]. Recent studies demonstrated that mitochondrial dysfunction in T cells promotes the release of highly diffusible inflammatory lipid hydroperoxides, which transfer oxidative stress to other intracellular organelles and through the bloodstream [5–7]. T lymphocytes of SLE patients exhibit persistent mitochondrial hyperpolarization (MHP), cytoplasmic alkalinization, increased ROI production, and diminished levels of intracellular glutathione [4, 7–10]. Increased production of nitric oxide has been identified as a cause of MHP and increased mitochondrial biogenesis; oxidative stress then affects signaling through the T cell receptor (TCR) [4].

In T cells from SLE patients and animal models of the disease, the main intracellular antioxidant glutathione is consumed and oxidized [11, 12]. Reversal of glutathione depletion by application of its amino acid precursor, N-acetylcysteine, decreases disease activity in lupus-prone mice [7, 11, 13]. Doherty and colleagues showed that O_2_ consumption in the peripheral blood lymphocytes (PBL) of SLE patients was increased. Furthermore, SLE PBL consumed more O_2_ upon in-chamber T cell activation, and N-acetylcysteine diminished O_2_ consumption [14]. These data indicate that SLE PBL exhibit increased O_2_ consumption that is inhibited by N-acetylcysteine, which may have therapeutic efficacy through reducing oxidative stress in SLE [14]. A clinical research showed that treatment combined with N-acetylcysteine and hydroxychloroquine, an antimalarial drug with ability to reduce the sensitivity of skin to UV exposure, could inhibit oxidative stress status and relieved the lupus activity [15]. Together, these data indicate that oxidative stress plays a key role in T cell dysfunction and the progression of lupus.

## 2. Oxidative Stress and Treg Cells

Regulatory T (Treg) cells are capable of modulating the function of effector T cells, maintaining immunologic homeostasis, and preventing autoimmunity [16]. Circulating Treg numbers decrease during disease flares in SLE patients, and the immune suppressive function of Treg cells in lupus is impaired [17–21]. The underlying reasons for this decrease in the number and function of Treg cells in SLE are not clear. Foxp3 is essential for the development and function of CD4^+^CD25^+^ regulatory T cells [22], and induction of the transcription factor Foxp3 can transform CD4^+^CD25^−^ naïve T cells into CD4^+^CD25^+^ regulatory T cells [22]. In addition, Treg cell activity can be induced in the peripheral immune system by the conversion of naïve CD4^+^Foxp3^−^ T cells into Foxp3^+^ T cells via transforming growth factor-beta (TGF-*β*) [23, 24]. In addition, interleukin- (IL-) 6 is a key cytokine that inhibits Foxp3 expression during Treg cell differentiation [24]. Previous studies have demonstrated that hyperoxia-induced stress and oxidative damage lead to an increase in IL-6, and, concordantly, increased production of IL-6 has been documented in SLE patients [24, 25]. Thus, oxidative stress-induced increased production of IL-6 may be the primary reason for the decrease in Treg cells in SLE patients.

Activation of the mammalian target of rapamycin (mTOR) has recently emerged as a key sensor of MHP and mediator of enhanced Ca^2+^ flux in lupus T cells [4, 26]. mTOR is a ubiquitous serine/threonine kinase that is a critical integrator of environmental stimuli and regulates signaling in T cells to influence homeostasis, differentiation, and activation [27, 28]. The effects of mTOR are cell type-specific and are elicited in response to stimulation by growth factors, hormones, and cytokines, as well as in response to internal and external metabolic products [29]. Recent observations have demonstrated that MHP and enhanced Ca^2+^ fluxing in T cells contribute to injury in lupus [30, 31]. In T cells, increased production of nitric oxide and MHP were identified as metabolic checkpoints upstream of mTOR activation [31, 32]. Additionally, mTOR controls the expression of the TCR-associated signaling protein CD4, and through increased expression of the endosome recycling regulator HRES-1/Rab4, mTOR mediates enhanced Ca^2+^ flux, skews the expression of tyrosine kinases in T cells, and blocks the expression of Foxp3 and the expansion of regulatory cells [31, 32].

Recently, additional work further demonstrated that mTOR controls the differentiation and functions of Treg cells, suggesting that its activity could be targeted to modulate Treg responses [27, 29]. Specifically, mTOR was identified as a component of two interacting complexes, mTORC1 and mTORC2, that regulate T cell lineage differentiation [33, 34]. Activation of mTOR delivers an obligatory signal for the proper activation and differentiation of effector CD4^+^ T cells, whereas, in Treg cell differentiation, the Akt-mTOR axis is a negative regulator of Treg cell [29, 33, 34]. Specifically, mTORC1 drives the proinflammatory lymphocyte subset expansion of T helper 1 (Th1) and T helper 17 (Th17) cells; mTORC2 favors the expansion of T follicular helper (Tfh) cells [35], which promote B cell activation and autoantibody production [29]. Both mTORC1 and mTORC2 inhibit the development of CD4^+^CD25^+^Foxp3^+^ Treg cells [29].

Furthermore, mTORC1 activity is high in human and murine Tregs [27, 29, 33, 36], and this activity restrains TCR and/or IL-2 stimulation-induced proliferation of Treg cells in vitro [36]. Rapamycin, a lipophilic macrolide antibiotic that regulates mitochondrial transmembrane potential and Ca^2+^ flux, has been used safely and effectively to treat lupus [31, 32, 37]. Rapamycin inhibits mTORC1 in Tregs and then promotes Treg cell expansion in SLE patients [38]. The effectiveness of rapamycin in murine and human SLE further supports the notion that mTOR is indeed a key mediator of autoimmunity in SLE. Therefore, understanding the mechanisms underlying the persistent MHP that leads to mTOR activation and enhanced Ca^2+^ flux is fundamental to understanding the pathogenesis of lupus [32]. These findings suggest that mTOR blockade may increase life expectancy via treatment and prevention of SLE inflammatory injury [7, 13, 29].

Another T cell biology regulator is leptin, which is a hormone derived from adipocytes. Leptin is closely related to adipokines, which are associated with oxidative stress and are reported to be overproduced in SLE patients [39, 40]. Previous data indicate that leptin signaling modulates T cell proliferation and preferential differentiation of Th1 cells over Th2 cells [41–43]. Recently, high levels of leptin receptor were detected in Treg cells. Interestingly, leptin was found to restrain Treg cell proliferation, as neutralization of leptin enhanced TCR and IL-2-induced Treg cell expansion [43–45]. Leptin receptor-deficient Treg cells also have increased proliferative responses linked to reduced mTOR activation [36, 41, 46, 47]. Thus, leptin sensing in Tregs is critical for dampening excessive mTOR activation and driving Treg proliferation [48]. Both mTOR inhibition and amino acid deprivation synergize with TGF-*β* to augment Foxp3 expression in vitro [27, 47, 48]. High leptin secretion has been reported in lupus patients, and thus, this secretion may be one of the key reasons underlying the induction of mTORC1-meditated inhibition of Treg cell differentiation, as well as proinflammatory Th17 cell and other effector T cell expansion in SLE [27, 49, 50]. Recent study showed that superoxide production is increased in all lymphocyte subsets of patients with antiphospholipid syndrome (APS), a special type of SLE. The oxidative stress was related to CD4^+^CD25^+^Foxp3^+^ Treg cell depletion in APS patients [51].

**Figure 1:**
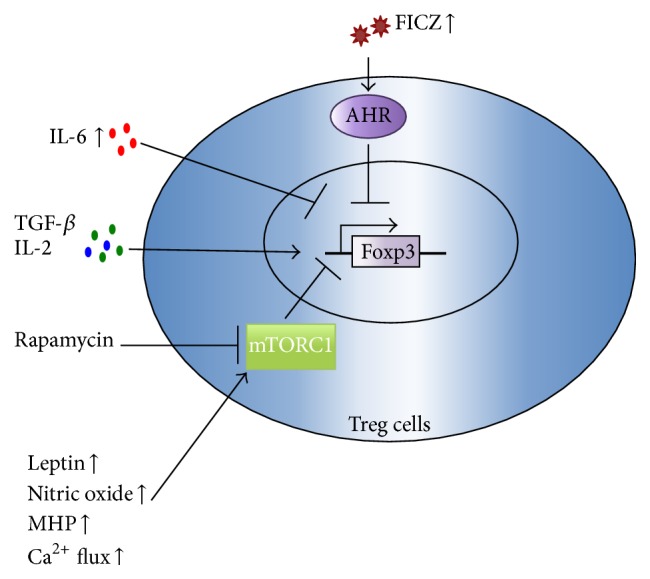
Oxidative stress and mTORC1-mediated Treg cell differentiation. TGF-*β* and IL-2 can induce CD4^+^Foxp3^+^ Treg cells expansion, and overproduction of IL-6 in SLE patients inhibits Foxp3 expression during Treg cell differentiation. Oxidative stress can promote increased production of leptin, nitric oxide, MHP, and enhanced Ca^2+^ flux in lupus T cells, which can result in overexpression of mTORC1, and then inhibit the development of CD4^+^CD25^+^Foxp3^+^ Treg cells. UV exposure-mediated increased production of FICZ can activate AHR signaling pathway and then inhibit Foxp3 expression in SLE. Rapamycin can inhibit mTORC1 in Tregs and then it promoted Treg cell expansion.

These data indicate that oxidative stress may contribute to the depletion of Treg cells in SLE patients, suggesting that antioxidation treatment may be an effective method to relieve lupus symptoms via improving Treg cell differentiation. Thus, strategies to enhance the number and function of Treg cells may benefit patients with SLE. Leptin and mTOR may also serve as effective intervention targets to relieve oxidative stress and improve the differentiation of Treg cells in SLE (Figure 1). In addition, Chinese herbs, or compounds isolated from natural herbs, also appear to be promising therapeutic agents for control of the oxidative stress noted in SLE patients.

## 3. Oxidative Stress and Th17 Abnormality

The Th17 lineage is a lineage of effector CD4^+^ T cells and is characterized by the production of IL-17 [52, 53]. Expansion of Th17 cells has been included in a growing list of autoimmune disorders [53, 54]. Our studies, as well as those of others, have demonstrated that Th17 cells play a key role in the pathogenesis of SLE [17, 18, 54]. Environmental factors, including exposure to UV radiation, infection, environmental pollution, and emotional changes, are believed to contribute to the increased prevalence of SLE and to aggravate lupus activity [55]. UV radiation exposure is among the environmental factors that have been studied the most with respect to association with SLE [55, 56]. While experimental studies show a significant immunomodulatory role for UV radiation, epidemiologic data describe its role in triggering SLE onset and patient deterioration [55–59]. To date, we understand that both UVB and UVA play a role in the pathogenesis of lupus erythematosus [58]. In the epidermis, UV radiation induces DNA damage, exposes nuclear antigens and photoinduced neoantigens at the cell surface, leads to an accumulation of apoptotic material, and induces several proinflammatory cytokines [58]. In the dermis, UV radiation triggers skin inflammatory cell infiltration [58]. UV radiation-induced signaling involves two major pathways: one that is initiated through the generation of DNA photoproducts in the nucleus and one that occurs independently of DNA damage and is characterized by cell surface receptor activation [58].

The aryl hydrocarbon receptor (AHR) is a ligand-dependent transcription factor best known for mediating the toxicity of dioxin [60]. AHR was discovered as a specific binding site for 2,3,7,8-tetrachlorodibenzo-p-dioxin (TCDD), an environmental toxin [61]. AHR detects not only pollutants, but many other environmental compounds as well, such as indoles and flavonoids of dietary origin and tryptophan metabolites that are generated by exposure to UV light or by bacteria of the intestinal microflora [62]. AHR expression is highly upregulated in Th17 cells following activation by both TGF-*β* and IL-6 and promotes IL-22 production and enhances Th17 development [63]. The ligand-activated transcription factor AHR is identified as a regulator of Treg and Th17 cell differentiation and plays a key role in both Treg cell development and Th17 cell differentiation [64, 65]. Intriguingly, two different high-affinity ligands for AHR activation, TCDD and 6-formylindolo[3,2-b]carbazole (FICZ), can play different roles in the differentiation of Th17 and Treg cells [64]. AHR activation by its ligand TCDD induces functional Treg cells that suppress experimental autoimmune encephalomyelitis (EAE) [61, 64]. Conversely, AHR activation by FICZ interferes with Treg cell development, promotes Th17 cell differentiation, and aggravates the severity of EAE in mice [64]. Thus, AHR regulates both Treg and Th17 cell differentiation in a ligand-specific fashion, constituting a unique target for therapeutic immunomodulation. Dorgham and colleagues demonstrated that propranolol, a potential lupus-inducing drug, caused stronger AHR activation in PBMCs of SLE patients than in those of controls [66]. This compound behaves like the prototypic AHR ligand FICZ, promoting IL-22, IL-8, and CCL2 secretion by T cells and macrophages [66]. These data indicate the AHR activation may play a key role in the pathogenesis of SLE.

AHR typically resides in the cytoplasm in complex with Hsp90 until the binding of its ligand triggers conformational changes, resulting in an exchange of Hsp90 for the nuclear translocation component ARNT [64, 67]. The endogenous AHR ligand FICZ may contribute to adverse physiological responses evoked by small natural and anthropogenic molecules, as well as by oxidative stress and light [68]. FICZ is formed from tryptophan in aqueous solutions upon exposure to UV and visible light and also in human skin cells exposed in vitro to UVB light [68, 69]. UVB, hydrogen peroxide (H_2_O_2_), and 3′-methoxy-4′-nitroflavone (MNF) promote the generation of FICZ and thereby give rise to prolonged AHR signaling [70, 71]. Intracellular formation of FICZ interacted with AHR and then results in translocation of AHR into the nucleus and induction of* CYP1A1* gene expression and elicits inflammation enlargement [64]. UVB radiation triggers AHR signaling by generating FICZ [62], and UVB irradiation-induced deterioration in SLE might be caused by the production of FICZ, AHR activation, and subsequent Th17 cell expansion [66, 69]. Thus, AHR-FICZ signaling is an integral part of the UVB stress response, and the AHR may, therefore, represent a target for therapeutic intervention in lupus.

**Figure 2:**
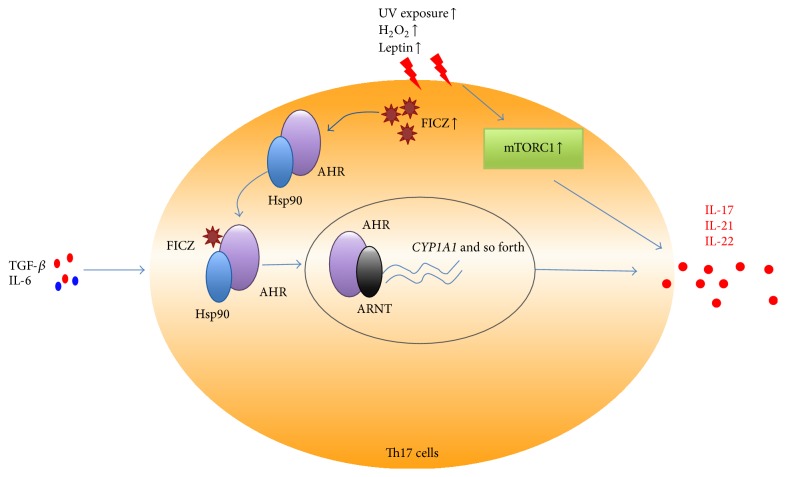
Oxidative stress and AHR-mediated Th17 cell differentiation. Increased UV exposure, H_2_O_2_, and leptin can promote the generation of FICZ, a natural ligand of AHR. AHR usually resides in the cytoplasm in complex with Hsp90. Interaction between AHR and ligands FICZ triggers conformational changes resulting in an exchange of Hsp90 for the nuclear translocation component ARNT and induces target genes expression such as Cyp1a1, triggering Th17 cell expansion and IL-17, IL-21, and IL-22 production. Oxidative stress can also elicit the activation of mTORC1, which then promotes Th17 cell differentiation and related cytokines production.

Based on the above discussion, we speculate that, in SLE patients, UV exposure can directly induce DNA damage, generation of DNA photoproducts, and apoptotic material, which consequently induces B cell activation and autoantibody production. Furthermore, UV exposure elicits AHR activation and the generation of FICZ, which is a high-affinity ligand for AHR activation and can further promote the differentiation of the Th17 cell population, thereby worsening the severity of lupus (Figure 2). Furthermore, the expansion of Th17 cells and overproduction of IL-17 may further aggravate lupus injury, as IL-17 can amplify the immune response by inducing the local production of chemokines and cytokines, recruiting neutrophils and monocytes, augmenting the production of autoantibodies, and aggravating the inflammation and damage of target organs, such as the kidney, in SLE.

Enhanced Ca2^+^ flux has been a hallmark characteristic in SLE patients and recent studies have demonstrated the involvement of calcium/calmodulin-dependent protein kinase IV (CaMK4) in the pathogenesis of SLE [72, 73]. CaMK4 can facilitate the recruitment of IL-17-producing cells to kidney of antiglomerular basement membrane antibody-induced glomerulonephritis (AIGN) mice, and Camk4-deficient mice displayed less glomerular injury and decreased infiltration by IL-17-producing CD4 T cells in kidney [74]. In addition, inhibition of CaMK4 could reduce IL-17 transcription through reduced activation of the AKT/mTOR pathway, which is known to enhance retinoic acid receptor-related orphan receptor- (ROR*γ*t-) mediated Th17 cell differentiation. Importantly, silencing CaMK4 in T cells from patients with SLE inhibited Th17 differentiation. Collectively, these data suggest that CaMK4 inhibition has potential as a therapeutic strategy for Th17-related SLE patients [75].

**Figure 3:**
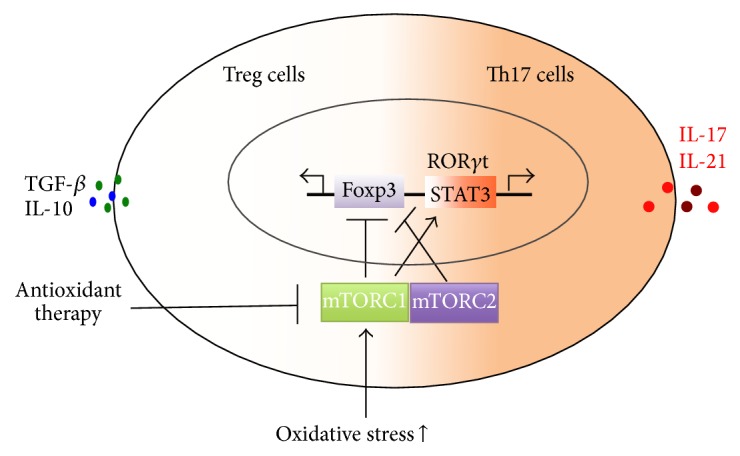
Oxidative stress and mTOR-mediated Th17/Treg imbalance in SLE patients. Oxidative stress can elicit the activation of mTORC1 and mTORC2. Both mTORC1 and mTORC2 can then inhibit Foxp3-mediated Treg cell development. mTORC1 promotes the expansion of Th17 cells via activating STAT3 and ROR*γ*t. And antioxidant therapy and mTOR blockade might be useful for regulating Th17 and Treg cell immune balance in SLE patients.

mTOR is identified as a component of two interacting complexes, mTORC1 and mTORC2, that regulate T cell lineage specification differentiation [29]. Oxidative stress in SLE can induce the activation of mTORC1; TORC1 signaling positively promotes Th17 cell differentiation via multiple mechanisms including the regulation of HIF-1 expression, STAT3 phosphorylation, and the nuclear translocation of ROR*γ*t [35, 76]. Both mTORC1 and mTORC2 inhibit the development of Treg cells; mTORC2 promotes the expansion of Tfh and Th2 cells [29, 35]. In this regard, antioxidant therapy and mTOR blockade promise to relieve the inflammatory injury via regulating immune balance in SLE (Figure 3).

## 4. Antioxidant Therapy in SLE

Our previous findings demonstrated that baicalin, a compound isolated from a Chinese herb, increased CD4^+^CD25^+^Foxp3^+^ Treg cell differentiation in vitro and that baicalin treatment protected lupus-prone MRL/lpr mice against nephritis and improved the Treg cells in vivo [77, 78]. Currently, baicalin is considered to be a potent antioxidative stress drug [79, 80]. Therefore, we hypothesized that baicalin may promote the differentiation of Treg cells via antioxidative stress effects; however, we have yet to test this hypothesis directly. Hydroxychloroquine, a drug often used in the treatment of SLE with ability to reduce sensitivity of the skin to UV exposure, can inhibit Th17 cell expansion and IL-17 production [81]. Our research group is working on the underlying mechanism whether hydroxychloroquine inhibits Th17 cell expansion via regulating AHR-FICZ signaling pathway in SLE patients. Resveratrol, a powerful antioxidant, possesses protective effects in pristane-induced lupus mouse model 82. Although resveratrol can inhibit mTOR pathway [82, 83], further study should be performed on whether resveratrol relieves lupus injury via inhibiting mTOR-mediated Th17 cell expansion. Antroquinonol, a purified compound and major effective component of* Antrodia camphorata* with antioxidant activities, has been proved to prevent the transformation of mild lupus nephritis into higher-grade nephritis in a murine lupus model [84]. Antroquinonol inhibited production of reactive oxygen species and nitric oxide and enhanced Treg cell suppression via increasing activation of nuclear factor E2-related factor 2 (Nrf2), which is referred to as the “master regulator” of the antioxidant response [84]. Epigallocatechin-3-gallate, the major bioactive polyphenol present in green tea with antioxidant and free radical scavenging activities, has been proved to prevent lupus nephritis development in mice via reducing proteinuria, renal function impairment, and improving renal injury [85]. Epigallocatechin-3-gallate has prophylactic effects on lupus nephritis that are highly associated with its effects of enhancing the Nrf2 antioxidant signaling pathway, decreasing renal NLRP3 inflammasome activation, and increasing systemic Treg cell activity [85]. A randomized, double-blind, placebo-controlled study has proved that N-acetylcysteine reduces disease activity by blocking mTOR pathway in T cells from systemic lupus erythematosus patients. N-acetylcysteine could reverse expansion of CD4^−^CD8^−^ T cells, stimulate Foxp3 expression in CD4^+^CD25^+^ T cells, and reduce anti-DNA production [13]. Altogether, these data suggest that antioxidant drug might be promising therapeutic methods for the treatment of SLE, and the specific therapeutic mechanism needs to be further studied.

**Figure 4:**
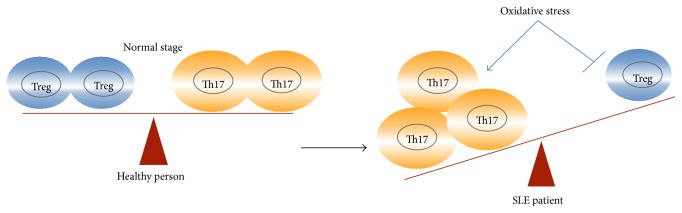
Oxidative stress and Th17/Treg imbalance in SLE patients. Under the normal stage, Th17 cells and Treg cells stay in a dynamic immune balance. Increased oxidative stress was induced in SLE patients, which can further induce and expand the proinflammatory Th17 cells and inhibit the anti-inflammatory Treg cell differentiation and aggravate autoimmune injuries.

Taken together, these data show that oxidative stress plays a key role in the pathogenesis of SLE. Oxidative stress can induce and aggravate SLE by linking environmental stimulation with immune imbalance. Oxidative stress contributed the Th17/Treg imbalance in SLE patients. Under the normal stage, Th17 cells and Treg cells stay in a dynamic immune balance, while oxidative stress was induced in SLE patients, which can further induce and expand the proinflammatory Th17 cells expansion and inhibit the anti-inflammatory Treg cell differentiation and aggravate autoimmune injuries (Figure 4). Above, we described the receptors and transcription factors that may offer key targets for the regulation of oxidative stress and immunity and thus for the treatment of SLE.
